# Social determinants that lead to poor knowledge about, and inappropriate precautionary practices towards, avian influenza among butchers in Kathmandu, Nepal

**DOI:** 10.1186/2049-9957-2-10

**Published:** 2013-06-05

**Authors:** Mohan Paudel, Bimala Acharya, Mandira Adhikari

**Affiliations:** 1 Health Right International, Kathmandu, Nepal; 2 Sahid Gangalal National Heart Center, Kathmandu, Nepal; 3 Population Service International, Kathmandu, Nepal

**Keywords:** Cross-sectional survey, Influenza in bird, Knowledge, Nepal, Social determinants

## Abstract

**Background:**

Avian influenza (AI) is a global public health threat. Understanding the knowledge that butchers have about it and the precautionary practices they take against it is crucial for designing future preparedness programs. This study aimed to identify the social determinants of knowledge and precautionary measures of AI among butchers in the Kathmandu district in Nepal.

**Methods:**

The study was based on a cross-sectional study design using structured interview questionnaires and checklists to observe social determinants and the precautionary measures of 120 butchers aged 15 years and above from the Kathmandu district.

**Results:**

The majority of the respondents were male (69.2%) and more than half (53.3%) were from the age group of 25–39 years (mean: 31.08, SD: ±9.82). Nearly two-thirds (61.3%) of the respondents had a ‘poor knowledge’, and the remaining had ‘some knowledge’, about AI. More than half (55.4%) of the respondents were in the category of displaying ‘poor practice’ towards AI and the remaining half were in the ‘satisfactory practice’ category. None of the respondents had ‘adequate knowledge’ or displayed ‘good practice’. The respondents in the >25 years of age group were less likely [OR 0.169; 95% CI (0.056-0.512)] compared to those in the <25 years age group to have a poor knowledge about AI; and the respondents with ‘primary education’ were more likely [OR 3.265; 95% CI (1.326-8.189)] to have a poor knowledge about AI as compared to those who had a secondary or above level of education. Respondents who did not know the correct definition of AI were more likely to follow poor practices [OR 4.265; 95% CI (1.193-15.242)]; and the respondents who did not know the risk groups associated with AI were also more likely to follow poor practices [OR 3.103; 95% CI (1.191-8.083)].

**Conclusion:**

This study points out the need to address butchers to improve their knowledge of, and more importantly their compliance with, the precautionary measures to prevent avian influenza.

## Multilingual abstracts

Please see Additional file 1 for translations of the abstract into the six official working languages of the United Nations.

## Background

Influenza is a global public health challenge. Whether in its zoonotic, seasonal epidemic, or pandemic forms, it can lead to mild to severe illness, and death [1]. Every year, seasonal influenza continues to cause significant mortality and morbidity on a global level. It is a highly infectious disease, and places the very young, the elderly and persons with chronic medical conditions at serious risks of infection and complications [2].

According to the 2005 World Health Organization (WHO) statement on ‘Avian influenza: responding to pandemic threat’, the great influenza pandemics of 1918–19 (Spanish flu H1N1) had caused illness in 25% of the total population and an estimated 20–40 million human deaths in a year [3]. The same report also mentioned that many of the deaths occurred in young and healthy persons in the age range of 15 to 35 years. Likewise, the pandemic flu of 1957–58 (Asian flu H2N2) and 1968–69 (Hong Kong flu H3N2) had each caused one to four million deaths, which led to huge social disruption and economic losses [4].

For several reasons, the highly pathogenic H5N1 virus is the greatest concern in present times [1,3,5]. Of all avian influenza (AI) viruses known to infect humans, H5N1 has caused the greatest number of very severe cases and the largest number of deaths. Moreover, H5N1 has the potential to trigger an influenza pandemic. In recent years, highly pathogenic AI A (H5N1) has caused unprecedented outbreaks in poultry in Asia and devastated the poultry industry [5,6]. In addition, the virus has also proved to be particularly difficult to control in poultry populations and is now considered endemic in parts of Southeast Asia [5]. Not only that, the virus managed to cross the species barriers resulting in severe illness and death in the human population [5]. Among 11 Southeast Asian countries, AI has a disproportionate impact with 39% of all cases and 52% of deaths occurring in just four countries (Bangladesh, Indonesia, Myanmar, and Thailand) [5]. From December 2003 to June 2012, 357 human deaths out of 605 human cases of H5N1 infection had been reported to the WHO from 15 countries with an average 60% case fatality rate [7].

In Nepal, the AI virus (H5N1) outbreak in poultry first occurred in 2009 in the Jhapa district. Since then, 21 outbreaks have been noted in poultry in 13 other Nepalese districts as reported to the World Organisation of Animal Health [8]. A total of 32,641 chickens and ducks died due to bird flu, and a further 56, 370 chickens and ducks, which were susceptible, were destroyed during these outbreaks. There was also a recent (2012) outbreak of AI on a poultry farm in the Bhaktapur district (an adjoining district located 15 kilometers east of Kathmandu, the capital city of Nepal) [9]. In this outbreak, there were 2,000 susceptible and 1,220 dead birds. According to the WHO estimation, if an influenza pandemic occurs in Nepal, the likelihood of human impact will be severe, and it is estimated that 6,400,000 people will be sick, 12,80,000-32,00,000 will have to seek outpatient care, 64,000- 704,000 will require hospitalization and 14,720-134,400 people will die [1].

The AI control project implemented by the Ministry of Health and Population (MOHP) Nepal had placed a particular emphasis on precautionary behaviors, knowledge, and attitude of high-risk groups in relation to AI [1].

The department of live stocks under the Ministry of Agriculture and Cooperatives (MOAC) and the department of health service under the MOHP Nepal have set the following preconditions for a butcher house: (i) there should be a separate butcher house for poultries and a separate one for other meat products, (ii) the butcher house should be neat and clean; there should be no stagnation of water, (iii) the wall of the butcher house should be cemented; there should be proper mechanisms for the disposal of waste, (iv) only clean equipment should be used and there should be hand washing facilities, (v) butchers have to use personal protective equipment, such as apron, boots, face mask, and goggles during slaughtering/de-feathering/slicing, (vi) the meat storage table/freeze/cupboard in the butcher house should be situated at least half a meter above the floor, well covered by a net and transparent glass to prevent from contamination, and (vii), a butcher house needs to be registered with the municipality office/local government office [10].

According to the provisions made by the 2007 ‘Bird Flu Control (With Update) Directives’ as approved by the cabinet of the Nepal Government, all poultry is to be culled within the circumference of three kilometers from the identified geographic location of the bird flu infection. The same provision further adds that in case of densely-populated areas and those places with numerous poultry areas, a technical team from the department of live stock service could make further assessment and declare the ‘infected zone’ to be even less than three kilometers away from the bird flu detected location [10,11].

Research on knowledge and existing practices is essential to direct preparedness and prevention programs in order to protect the vulnerable groups, such as butchers, who are involved in slicing, de-feathering, and slaughtering of the poultry. Except for one study from the western region of Nepal [12], none of the studies have reported on the knowledge and practice of AI, and only one study has previously reported on knowledge and practice of AI among poultry farm workers of Nepal. Therefore, this study aimed to identify the knowledge about, and practice of, precautionary measures against bird flu among butchers in the Kathmandu district in Nepal.

This study built on the foundation of the Health Belief Model. The knowledge and the adoption of precautionary practices is influenced by a range of factors: perceived susceptibility and severity of AI, socio-demographic characteristics, and perceived benefits of preventive measures. Combined, these precautionary practices ultimately lead to a decreased risk of human transmission [13].

## Methods

### Participants and procedure

A cross-sectional study was conducted between June and July 2012. Face-to-face interviews were conducted using a structured questionnaire with 120 butchers aged 15 years and above. The observation checklist was used to observe the onsite practices related to precautionary measures against avian influenza (AI).

The Kathmandu district was purposively chosen because it is one of the districts with a high population density, thus making it favorable for person-to-person transmission of the infection. Furthermore, it is a district with the highest number of reported butcher houses (N = 400) in Nepal. It is also one of the 26 high-risk districts for bird flu infection in the country [14]. According to the 2011 National Census Report, Kathmandu has a population of 1,740,977 and the highest population density – 4,408 per square kilometer – in the country (Nepal has a population density of 181 per square kilometer) [15]. The high-risk groups, especially poultry farm workers including butchers, are the ‘bridging population’, a cross-species sharing viruses and possessing the potential to spread the disease to locals in their regions [12].

To prepare the sampling frame, a list of butcher houses in the Kathmandu district was obtained from the Nepal Machha Masu Byabasayi Sangh (Nepalese Association of Fish, Poultry and Meat Workers), the established professional organization of the meat and poultry sector in Kathmandu. A list of butcher houses registered in this organization (the umbrella organization of butchers) was obtained. As of our study date, a total of 400 butcher houses were registered. This was the only available sample frame at the time of the survey as such a list was not available from the government livestock authority or the metropolitan office. Registration to the organization is voluntary, therefore, it is likely that some of the butcher houses were not registered. However, to our knowledge, this organization includes most of the butcher houses from the study area.

With a population size of 400, a hypothesized % frequency of outcome factor in the population (p): 50% (±10%), confidence limits 99% (absolute +/− %) (d):10%, and a design effect of 1.0 (random sampling), we obtained 118 as the required sample size. However, we interviewed 120 respondents. We selected the participants by using a systematic random sampling so that all individuals had an equal opportunity to be selected and also to make sure that all study areas were included in the sample. Inclusion criteria for the participants were: (i) only one person from one butcher house, (ii) more than 15 years of age, (iii) available during the time of the interview, and (iv) it was the person approached first if there was more than one butcher in the shop. A person was excluded even if he or she met the above criteria but was working as a cashier in the butcher house and/or not involved in slaughtering, de-feathering, or slicing of the poultry.

### Interview questionnaire

The interview was based on questionnaires adopted from the published sources of similar settings in Nepal and Italy [12,16]. Pre-testing of the questionnaires was done in 10% of the sample size in the Lalitpur district, the adjoining district of Kathmandu. Necessary modifications (added others, don’t know category, added multiple responses, and formatted the general layout of the questionnaire form) were drawn up based on feedback. The observation checklist was developed consulting a previous study done in Rupandehi, Nepal on precautionary measures towards AI among poultry farm workers, as well as the report published by the Nepal Avian Influenza Control Project (AICP) [1,12].

### Definition of variables

Knowledge and precautionary measures were the two main outcome variables included in this study. The responses regarding the respondents hearing about AI, high-risk groups for getting the bird flu, modes of transmission, signs and symptoms, and precautionary measures of AI prevention were considered as constituting knowledge about AI. To measure knowledge, the scoring system from a previous Thai study was adopted [14]. The categories were ‘adequate’ (29–37 points i.e. score above 80%), ‘fair’ (15–28 points i.e. scoring 45–79%), ‘poor’ (1–14 points i.e. scoring < 45%), and ‘no knowledge’ (0 points). The total knowledge score was 37. For our further analysis, we categorized knowledge into ‘poor’ (<14 points, <45%) and ‘satisfactory’ (>14 points, >45%) as binary variables.

In the structured interview questionnaires, respondents were asked about what they considered to be the ‘definition of AI’ with the following options: (i) disease of chickens and ducks, (ii) an infectious communicable disease that can affect all species of birds, (iii) disease of pigs, (iv) disease of man, and (v) don’t know. The respondents who chose option ‘(ii)’ were marked as having the ‘correct factual knowledge’. All other options were misconceptions regarding the ‘cause’ and were therefore marked as ‘incorrect’. The other questions asked relating to knowledge were ‘cause of bird flu’, ‘mode of transmission in humans’, ‘risk factors associated with bird flu’, and ‘knowledge about precautionary measures’. The responses were coded as ‘correct’ and ‘incorrect’ based on the respondents’ knowledge regarding the scientific/biomedical definitions.

The basic protection measures, specifically the use of masks, gloves, apron and boots, hand washing after touching raw meat, presence of a hand washing facility, and cleaning of utensils in the butcher house were considered as being ‘good practice’, and were initially labeled as ‘good’ (7–9 score), ‘satisfactory’ (4–6), and ‘poor’ (1–3) based on a scoring system done from a Thai study [17]. For our further analysis, we re-categorized this into binary variables ‘poor’ (<4 score) and ‘satisfactory’ (>4 score). There were nine total statements to measure precautionary practice; one score was assigned per precautionary statement.

The study had first categorized religion into Hindu, Muslim, Buddha, Kirat, and Christian. For further analysis, ‘Hindu’ and ‘non-Hindu’ were specified to ensure sampling adequacy in each category. We used the ethnic categorical definition of the Nepal Health Management Information System, which has categorized ethnicity into six different groups. However, for further analysis, we categorized it into: (i) Brahman/Kshetri/Newar, and (ii) Janajati/Minority and Terai caste (this includes Muslim, the Terai caste, and *Janastis* – the indigenous) [18,19]. Work duration was recorded in years. For the analysis, we categorized it into: (i) more than five years and (ii) less than five years. As the distribution was segregated by literacy status (and this can skew knowledge), we recoded the education attainment for our further analysis as: (i) primary (completing grade 8 and/or below) and (ii) secondary and above (completing grade 9 and/or above).

### Statistical analysis

Descriptive statistics were used to describe the socio-demographic characteristics of the respondents. The chi-square test was used to examine the relationship between categorical socio-demographic and outcome variables (knowledge and precautionary measures). The stepwise backward logistic regression method was used to obtain the final multiple logistic regression model for knowledge and practice. P value <0.05 was considered statistically significant. Data analysis was conducted using the Statistical Package for Social Sciences (version 19.00).

The study protocol and data collection tool was approved by the Research Ethics Committee of the Institute of Medicine at Tribhuvan University, Nepal.

## Results

### Knowledge and precautionary measures related to avian influenza (AI)

The majority of the respondents were male (69.2%), Hindus (88.3%), and married (80%). The majority (53.3%) of the respondents were in the age group 25–39 years (mean: 31.08 years, SD:±9.82). One in five (19.2%) respondents were illiterate and four in ten (41.7%) had been working as a butcher for more than five years. The majority of the respondents (78.3%) were from the upper caste *(Brahmin)* and 88.3% were Hindu. The majority of the respondents (94%) did not have any previous training related to meat hygiene. Nearly three-quarters (72.5%) of the respondents were the owners of the butcher houses where they worked (see Table 1).

**Table 1:** Socio-demographic characteristics of the respondents

**Variable**	**Number**	**Percentage**
**Sex**		
Female	37	30.8
Male	83	69.2
**Age**		
<25	31	25.8
25-39	64	53.3
40 and above	25	20.8
**Caste**		
Brahman/Kshettri	95	78.3
Minority, Terai Caste, and Janajati	25	21.7
**Marital Status**		
Married	96	80
Unmarried	24	20
**Religion**		
Hindus	106	88.3
Non-Hindus (Buddhism, Muslim, Christian)	14	11.7
**Educational Status**		
Illiterate	23	19.2
Primary	53	44.2
Secondary and above	44	36.6
**Occupational Status**		
Owner	87	72.5
Paid employee	33	27.5
**Work Duration**		
<5 years	70	58.3
>5 years	50	41.7
**Training related to meat hygiene**		
Yes	7	5.8
No	113	94.2

The study revealed that nearly two-thirds (61.3%) of the respondents had a poor knowledge (score 0–14), and the remaining had some knowledge (score 22–28), about AI. None of the respondents had adequate knowledge (score > 28) (see Table 2). One in ten respondents had ‘no knowledge’ about a single personal protective measure. The use of aprons (76.1%) was the most common precautionary measure, followed by hand washing with soap and water after touching raw meat (60.5%). Nearly half (40%) knew about the protective capacity of gloves, 43.3% mentioned face masks as an option, and only a few knew about the special boots (12.8%) and goggles (2.7%) to use (see Table 3).

**Table 2:** Knowledge level of the respondents

**Knowledge level (Total Score – 37)**	**Number**	**Percent**
Satisfactory knowledge (score 22–28)	47	39.2
Poor knowledge (score 0–14)	73	60.8
**Total**	120	100.0

**Table 3:** Knowledge of the respondents about AI

**Knowledge variables**	**Number**	**Percentage**
**Definition of bird flu (n = 120)**		
Disease of chicken and ducks	83	69.2
Infectious communicable disease that can affects all species of birds	31	25.8
Disease of pigs	1	0.8
Disease of humans	2	1.7
Don’t know	3	2.5
**Cause of bird flu (n = 120)**		
Virus	34	28.3
Transportation of chickens and ducks from bird flu infected areas	16	13.3
Contact with migratory wild birds	10	8.3
Don’t know	60	50.0
**Mode of Transmission (n = 91)**^**a**^		
Contact with secretions of infected poultry	32	20.5
Eating chicken that is not properly cooked	76	48.7
Eating eggs that are not properly cooked	12	7.7
Unsafe handling of sick and dead poultry	7	4.5
Not washing hands with soap and water after handling poultry and raw meat	13	8.3
**Risk Group (n = 104)**^**a**^		
Butchers	65	37.8
Poultry workers	92	53.5
Person preparing poultry for consumption	5	2.9
Person handling carcasses of infected poultry	10	5.8
**Knowledge of precautionary measures (n = 109)**^**a**^		
Washing hands with soap and water after touching raw meat	66	19.6
Use of apron	83	24.7
Use of gloves	48	14.3
Use of face mask	52	15.5
Use of boots	14	4.2
Use of goggles	3	0.9
Cleaning of cutting utensils and surface	43	12.8
Proper disposal of waste materials	27	8.0

a: multiple responses.

Regarding the practice level of the respondents, this study found that slightly over half (55.4%) belonged to the category of displaying poor practice (score 1–3) and the other half (44.6%) demonstrated satisfactory practice (score 4–6). None of the respondents demonstrated good practice (score > 6) (see Table 4).

**Table 4:** Practice level of the respondents

**Practice (Total score = 9)**	**Number**	**Percent**
Satisfactory practice (4–6 score)	37	44.6
Poor practice (1–3 score)	46	55.4
**Total**	83	100.0

### Factors associated with having knowledge about AI

The association of significant independent variables in the chi-square test was further investigated by performing a multiple logistic regression. The age, literacy, and highest school level (educational attainment) of respondents were significantly associated with having knowledge about AI during univariate analysis (see Table 5). On subsequent logistic regression analysis, age and education attainment were the two most significant determinants of knowledge about AI. The respondents of the >25 years of age group were less likely [OR 0.169; 95% CI 0.056-0.512)] to have a poor knowledge of AI than the <25 age group. The respondents completing primary or below education were more likely [OR 3.295; 95% CI (1.326-8.189)] to have a poor knowledge than the respondents completing secondary or above level of education. The study reported that religion was not a significant variable, however, it was found significant in the unadjusted analysis (see Tables 6 and 7).

**Table 5:** Compliance with precautionary measures (N = 83)

**Precautionary measures compliance**^**a**^	**Response**	
	**Frequency**	**Percent**
Use of apron	58	20.9
Use of face mask	1	0.4
Use of gloves	0	0.0
Use of boots	3	1.1
Protect eyes (use of goggles)	1	0.4
Wash hands with soap and water after touching raw meat	22	7.9
Clean cutting utensils and surface	75	27.0
Proper disposal of waste materials	64	23.0
Presence of hand washing facilities	54	19.4

a: multiple response.

**Table 6:** Association of socio-demographic characteristics with knowledge level

**Factors**	**Categories**	**Satisfactory knowledge (39.2%)**	**Poor knowledge (60.8%)**	**Chi-square test (p value)**
**Age**	<25 years	5 (16.1)	26 (83.9)	0.007*
	25-39 years	32 (50.0)	32 (50.0)	
	40 and above years	10 (40.0)	15 (60.0)	
**Ethnicity**	Brahmin/Chhetri	40 (42.6)	54 (57.4)	0.148
	Janajati and Minority	7 (26.9)	19 (73.1)	
**Marital status**	Married	41 (42.7)	55 (57.3)	0.112
	Unmarried	6 (25.0)	18 (75.0)	
**Sex**	Female	11 (29.7)	26 (70.3)	0.157
	Male	36 (43.4)	47 (56.6)	
**Occupation**	Owner	37 (42.5)	50 (57.5)	0.221
	Employee	10 (30.3)	23 (69.7)	
**Literacy**	Literate	46 (47.4)	51 (52.6)	<0.001*
	Illiterate	1 (4.3)	22 (95.7)	
**Education attainment**	Primary	17 (32.1)	36 (67.9)	0.001*
	Secondary and above	29 (65.9)	15 (34.1)	
**Religion**	Hindu	45 (42.5)	61 (57.5)	0.042*
	Non-Hindu	2 (14.3)	12 (85.7)	

*statistically significant at p < 0.05.

**Table 7:** Factors associated with knowledge about AI

**Factors**	**Satisfactory knowledge N (%)**	**Poor knowledge N (%)**	**Crude odds ratio**		**Adjusted odds ratio**	
			**OR**	**95% CI**	**aOR**	**95% CI**
**Age**			p = 0.011		p = 0.006	
<25 Years	5 (16.1)	26 (83.9)	1.00		1.00	
25-39 Years	32 (50.0)	32 (50.0)	0.192	0.066-0.564	0.169	0.056-0.512
40 and above Years	10 (40.0)	15 (60.0)	0.288	0.083-1.004	0.182	0.47-0.698
Education attainment			p = 0.001		p = 0.003	
Secondary and above	29 (65.9)	15 (34.1)	1.00		1.00	
Primary	17 (32.1)	36 (67.9)	4.064	1.751-9.572	3.295	1.326-8.189
**Religion**			p = 0.059		p = 0.129	
Hindu	45 (42.5)	61 (57.5)	1.00		1.00	
Non-Hindu	2 (14.3)	12 (85.7)	4.426	0.944-20.765	3.673	0.683-19.66

-2loglikelihood = 129.977, df = 4.

Independent variables entered in initial model: age, literacy status, and religion.

### Factors associated with precautionary practices against AI

This study found that none of the variables were significantly associated with good practice (see Table 8). Knowledge about the cause of AI, risk groups, and transmission were found to be significantly associated with the precautionary measures in the univariate analysis (see Table 9). On subsequent regression analysis, it was demonstrated that the respondents who did not know the correct definition of AI were, in turn, more likely to demonstrate poor practices [OR 4.265; 95% CI (1.193-15.242)], and those who did not know about the risk groups of AI were also more likely to demonstrate poor practices [OR 3.103; 95% CI (1.191-8.083)] as well (see Table 10).

**Table 8:** Association of socio-demographic characteristics with precautionary measures

**Factors**	**Categories**	**Satisfactory practice N (%)**	**Poor practice N (%)**	**Chi-square test (p value)**
**Sex**	Female	12 (42.9)	16 (57.1)	0.822
	Male	25 (45.5)	30 (54.5)	
**Age**	<25	6 (37.5)	10 (62.5)	0.395
	25-39	21 (42.0)	29 (58.0)	
	> = 40	10 (58.8)	7 (41.2)	
**Marital status**	Married	31 (44.9)	38 (55.1)	0.887
	Unmarried	6 (42.9)	8 (57.1)	
**Religion**	Hindu	32 (43.8)	41 (56.2)	0.713
	Non-Hindu	5 (50.0)	5 (50.0)	
**Ethnicity**	Brahmin/Chhetri	27 (42.2)	37 (57.8)	0.421
	Janajati and Minority	10 (52.6)	9 (47.4)	
**Literacy status**	Literate	33 (49.3)	34 (50.7)	0.079
	Illiterate	4 (25.0)	12 (75.0)	
**Education attainment**	Primary	15 (41.7)	21 (58.3)	0.181
	Secondary and above	18 (58.1)	13 (41.9)	

**Table 9:** Association of knowledge variables with precautionary measures

**Factors**	**Categories**	**Precautionary measure**		**Chi-square test (p value)**
		**Satisfactory practice N (%)**	**Poor practice N (%)**	
Definition of bird flu	Correct	13 (76.5)	4 (23.5)	0.005
	Incorrect	24 (36.4)	42 (63.6)	
Cause of AI	No	23 (37.7)	38 (62.3)	0.036
	Yes	14 (63.6)	8 (36.4)	
Risk group	No	12 (28.6)	30 (71.4)	0.003
	Yes	25 (61.0)	16 (39.0)	
Transmission	Cannot transmit	4 (19.0)	17 (81.0)	0.006
	Can transmit	33 (53.2)	29 (46.8)	
Aware of signs of AI in chickens	Yes	23 (52.3)	21 (47.7)	0.134
	No	14 (35.9)	25 (64.1)	
Aware of AI signs and symptoms in humans	Yes	10 (50.0)	10 (50.0)	0.576
	No	27 (42.9)	36 (57.1)	

**Table 10:** Factors associated with precautionary measures

**Factors**	**Poor practice N (%)**	**Satisfactory practice N (%)**	**Crude odds ratio**		**Adjusted odds ratio**	
			**OR**	**95% CI**	**aOR**	**95% CI**
**Definition of bird flu**			p = 0.006		p = 0.026	
Incorrect	42 (23.5)	24 (36.4)	5.687	1.666-19.415	4.265	1.193-15.242
Correct	4 (63.6)	13 (76.5)	1.00		1.00	
**Cause of AI**			p = 0.040		p = 0.258	
No	38 (62.3)	23 (37.7)	2.891	1.052-7.949	1.864	0.634-5.485
Yes	8 (36.4)	14 (63.6)	1.00			
**Risk group**			p = 0.004		p = 0.020*	
No	30 (71.4)	12 (28.6)	3.906	1.561-9.778	3.103	1.191-8.083
Yes	16 (39.0)	25 (61.0)	1.00		1.00	
**Transmission**			p = 0.010		p = 0.216	
Cannot transmit	17 (81.0)	4 (19.0)	4.836	1.460-16.025	2.212	0.616-8.528
Can transmit	29 (46.8)	33 (53.2)	1.00	1.00	1.00	

*statistically significant at p < 0.05,-2loglikelihood ratio: 99.572; df: 2.

Independent variables entered into initial model: definition of bird flu, causes of AI, risk group, and mode of transmission.

## Discussion

Nepal experienced its first avian influenza (AI) outbreak in backyard poultry in 2009 in the Jhapa district. Until now, 21 more outbreaks have been reported in backyard and commercial poultry in 13 districts of Nepal [12]. Aside from huge social and economic disruptions, the WHO estimates that the likelihood of human impact results in over three to six million cases of disease and deaths, with 14,720 to 134,400 people seeking outpatient care and hospitalization [1].

Nepal experienced another outbreak in 2012 in the Bhaktapur district, in which about 2,000 birds died as reported by the Department of Live Stock Service, Lalitput to the World Organization of Animal Health [9]. Considering the likelihood of this catastrophic scenario, it would be effective to know the existing knowledge and appropriate prevention measures related to AI in order to try prevent AI cases. This study, therefore, aimed to identify the social determinants of poor knowledge about, and inappropriate precautionary measures towards, AI among butchers.

This study found that a substantial majority of the respondents were not following the recommended personal protective measures. Use of aprons was the most common precautionary measure according to the respondents. The knowledge about protective boots (12.8%) and goggles (2.7%) was surprisingly poor. An important finding was that a smaller number (43%) of the respondents valued face masks as a protective measure. It is likely that butchers might have perceived themselves at being at a lower risk. These findings about precautionary measures are similar to findings from a previous study conducted among poultry workers in the Rupandehi district of Nepal [12]. But, that particular study revealed a higher percentage of knowledge about hand washing (88.8%) and use of gloves (68.8%) than this study. This discrepancy might be because the previous study was conducted shortly after the first outbreak of AI in Nepal in 2009, which resulted in government-induced mass media campaigns focusing on hand washing as the key message during that first outbreak.

This study found that the literacy level (educational attainment) and the age of the respondent were significantly associated with knowledge about precautionary measures of bird flu. One study conducted in Thailand also revealed that education is a major determinant of having a higher level of knowledge [17]. The higher education level could have increased the level of exposure to mass media (print and electronic media), and also may have contributed to an increased perceived risk. The educated individuals have more chances to get information about AI through newspapers, radio, television, and the internet.

The current study found that the younger age groups (<25 years of age) had a lower knowledge about AI than the older age groups. The older respondents were likely to work longer in their profession than the younger ones. Due to longer work duration, they might have been exposed to, and attended, different orientation, media statements, and person-to-person discussions about the risk and precautionary measures associated with AI. They were also more likely to attend meetings and discussion programs of the poultry business-related associations, which are major stakeholders in bird flu control measures during the outbreaks. On the other hand, less experience might be associated with having less exposure to knowledge about work-related occupational hazards. The association of age was not found to be significant in the previous Thai study [17].

The present study found a poor compliance with precautionary measures. Only 26% of the respondents practiced hand washing with soap and water, 3.6% used boots, and none of the respondents used gloves. Use of various personal protective equipment (PPE) and sanitation measures are the part of the comprehensive response to prevent and control AI. Most of the human cases of AI were caused due to contact with infected poultry [20]. Contact with body surface (feet, hands, and face) increases the likelihood of getting an infection. Contrary to the present study, a previous study done in Nepal found a better compliance with personal protective measures [12]. The current study included the respondents who were from small-scale butcher houses, however, in the previous Nepalese study, many of the respondents were from commercial farms. Commercial farms are more likely to provide PPE. The present study observed the respondents’ practice in actual settings, whereas the previous Nepalese study collected practice responses through interviews. Overall, the current findings of poor compliance with the recommended precautionary practices could also be attributed to the poor legal enforcement that exists in relation to the conditions that are required to be met by the butcher houses regarding precautionary measures.

This study found a statistically significant relationship between knowledge and practices (see Tables 9 and 10). This finding is also consistent with the previous Nigerian and Nepalese studies [12,21]. Knowledge might have developed awareness and thus compliance with healthy practices; awareness might have caused respondents to perceive the threat of AI more readily. As we know from the Health Belief Model, the perceived threat/susceptibility of a risk supports the development of healthy habits [13].

The current study clearly pointed out that the ‘respondents <25 years’ and ‘butchers with primary education’ should be especially targeted with educational activities relating to AI; these groups had markedly poor knowledge about AI. The relatively low knowledge about personal protective measures has alerted that there is an immediate need to focus on promoting the use of PPE. National and international guidelines have unequivocally suggested the use of PPE to aid in the protection against AI threat [1,22]. Involvement of the poultry and butchers’ associations in enforcing PPE should be considered as being of utmost importance in future programs.

Studies conducted in different parts of the world have shown implications of knowledge and precautionary behaviors [17,23-25]. One qualitative study in Bangladesh found that poultry raisers recognized the AI transmission from poultry to poultry, but not from poultry to humans, and it also revealed that they usually kept sick poultry under the beds. Moreover, if the sick poultry did not recover, they slaughtered it to consume or sell [26]. Another qualitative study conducted in four different countries (Hong Kong, Guangzhou, Vietnam, and Thailand) explored HPAI as a periodic natural disease, thus being of little concern. Poor hygiene in husbandry practices and uncontrollable external explanations for vulnerability factors e.g. weather, seasonal changes, bird migration and pollution, were discussed by the respondents in this study [27]. In addition, one cross-sectional study conducted in Afghanistan in the general population identified a differing mean knowledge, attitude, and practice (KAP) scores according to socio-economic quintiles; it was higher in provinces previously exposed to Information Education Communication campaigns [28]. Furthermore, one Italian study showed the evident role that perceived knowledge has on compliance with the precautionary practices of AI [16]. The Italian study found that compliance with hygienic practices was more likely to be successful by those who perceived a higher risk of contracting, by those who knew that washing hands with soap before and after touching raw poultry meat and that using gloves is hygienic practice to avoid spreading of the virus through food, by those who knew about the modes of transmission and common vehicles, and by those who received information by health professionals and scientific journals.

As this study found that almost all respondents (95%) didn’t have training that increases the knowledge and skills to practice healthy butchering, slicing, and de-feathering, veterinary departments, including the departments of epidemiology under the government’s health division, should implement regulations regarding the need to train those working in butcher houses.

### Limitations and recommendations for further studies

This study is the first study to explore the knowledge about, and precautionary measures against, AI among the butchers in Nepal. Findings from this study will be useful for the AI prevention and control sections of the veterinary department. However, the relatively small sample size is one of the major limitations of this study. Further, the sample size of the respondents to explore the compliance of precautionary measures was also small. These might have underestimated the effects of independent variables on the outcome variables. This study, being a cross-sectional study, does not show causal relation, however, it does demonstrate the association between socio-demographic variables, and knowledge and practice. An intervention study to look at the feasibility to promote safe precautionary practices, especially among high-risk groups, is essential [1,22]. It would also be useful to explore the prevailing challenges for prevention and preparedness among high-risk groups (poultry workers and butchers) by using qualitative techniques [29-31].

## Conclusion

This study highlighted the critical gap that exists in having knowledge about AI, and its compliance related to its precautionary measures among butchers. Nearly two-thirds (61.3%) of the respondents had ‘poor knowledge’ about AI. Regarding the precautionary measures, more than half (55.4%) of the respondents demonstrated ‘poor practice’. The study draws attention to promote better knowledge for adopting recommended precautionary measures to prevent AI. Stakeholders are required to consider and target butchers in future prevention and preparedness programs.

## Competing interests

The authors wish to declare that they have no competing interests.

## Authors’ contributions

MP and BA were involved in conceiving the study, pre-testing, and data collection. MA contributed to the analysis. All authors were involved in writing the manuscript, reviewing it, and agreed on co-authorships. All authors read and approved the final manuscript.

## Author’s information

MP holds a Joint Masters in Sustainable Regional Health Systems from consortium universities: Deusto University in Spain, Corvinus University in Hungary and Vilnius University in Lithuania. He is the Monitoring and Evaluation Coordinator at HealthRight International, Nepal. BA holds a Bachelor of Nursing from Tribhuvan University of Nepal and has worked as a Maternal and Child Health Supervisor between 2008–2010 in Medical Emergency Relief International (MERLIN). MA is currently working at Population Services International. She holds a Bachelor of Public Health from Purbanchal University of Nepal.

## Supplementary Material

Additional file 1 Multilingual abstracts in the six official working languages of the United Nations. Click here for file
