# Supplementary material for: Social determinants that lead to poor knowledge about, and inappropriate precautionary practices towards, avian influenza among butchers in Kathmandu, Nepal
Source: Infect Dis Poverty. 2013 Jun 5;2:10. doi: 10.1186/2049-9957-2-10 (PMC3710200; doi:10.1186/2049-9957-2-10)
Supplement: Additional file 1 — Multilingual abstracts in the six official working languages of the United Nations. [file 2049-9957-2-10-S1.pdf]

Translation of the abstract into the six official working languages of the United Nations

المحددات الاجتماعية التي تؤدي إلى ضعف المعرفة بشأن، والممارسات الاحترازية غير الملائمة نحو، أنفلونزا الطيور بين الجزارين في منطقة كاتماندو، نيبال

موهان بوديل وبيمالا أشاريا وماندير أدهيكاري

ملخص

معلومات أساسية: تعد أنفلونزا الطيور تهديدًا للصحة العامة العالمية. ولذا فإن فهم المعرفة التي لدى الجزارين حول هذا الموضوع والممارسات الاحترازية التي يقومون بها لمقاومتها أمر ضروري لتصميم برامج الاستعداد للمستقبل. تهدف هذه الدراسة إلى التعرف على المحددات الاجتماعية للمعرفة والتدابير الاحترازية المتعلقة بأنفلونزا الطيور بين الجزارين في منطقة كاتماندو في نيبال.

الطرق: استندت الدراسة إلى تصميم دراسة مستعرضة باستخدام الاستبيانات بالمقابلات الشخصية والقوائم المرجعية المنظمة لرصد المحددات الاجتماعية والتدابير الاحترازية لدى 120 جزارا تتراوح أعمارهم من 15 عامًا وما فوق من منطقة كاتماندو.

النتائج: كانت غالبية المشاركين في الاستبيانات من الذكور (69.2٪) وكان أكثر من نصفهم (53.3٪) من الفئة العمرية بين 25-39 عامًا (يعني: 31.08، بانحراف معياري: ±9.82). كان ما يقرب من ثلثي (61.3٪) المشاركين في الاستبيانات لديهم 'معرفة ضعيفة'، والباقي كان لديهم 'بعض المعرفة'، حول أنفلونزا الطيور. وكان أكثر من نصف (55.4٪) المشاركين في الاستبيانات في الفئة التي تُظهر 'ممارسة سيئة' فيما يتعلق بأنفلونزا الطيور، بينما كان النصف المتبقي في فئة 'الممارسة المرضية'. ولم يحظ أي من المشاركين في الاستبيانات بـ 'المعرفة الكافية' أو يُظهر 'الممارسة الجيدة'. كان المشاركون في الاستبيانات من الفئة العمرية الأقل من 25 عامًا أقل عُرضة [نسبة الرجحان 0.169، 95٪ فاصل الثقة (0.056-0.512)] مقارنة بأولئك الموجودين في الفئة العمرية الأكبر من 25 عامًا، لامتلاك معرفة ضعيفة بشأن أنفلونزا الطيور، أما المشاركون في الاستبيانات الحاصلون على 'التعليم الابتدائي'، فكانوا أكثر عُرضة [نسبة الرجحان 3.265، 95٪ فاصل الثقة (1.326-8.189)] لامتلاك معرفة ضعيفة بشأن أنفلونزا الطيور بالمقارنة مع أولئك الذين كانوا في مستوى التعليم الثانوي أو أعلى. كان المشاركون الذين لم يعرفوا التعريف الصحيح لأنفلونزا الطيور أكثر عُرضة لاتباع الممارسات السيئة [نسبة الرجحان 4.265، 95٪ فاصل الثقة (1.193-15.242)]، وكان المشاركون الذين لا يعرفون الفئات المعرضة للمخاطر المرتبطة بأنفلونزا الطيور أكثر عُرضة لاتباع ممارسات سيئة أيضًا [نسبة الرجحان 3.103، 95٪ فاصل الثقة (1.191-8.083)].

الخلاصة: تشير هذا الدراسة إلى ضرورة مخاطبة الجزارين لتحسين معرفتهم بالتدابير الاحترازية لمنع انفلونزا الطيور، والأهم من ذلك تحسين امتثالهم لهذه التدابير.

Translated from English version into Arabic by Muhammad Assayed, through

TRANSLATORS
WITHOUT BORDERS

尼泊尔加德满都屠宰者中导致对禽流感知识贫乏和不适当的预防实践的社会决定因素

Mohan Paudel, Bimala Acharya and Mandira Adhikari

摘要：

引言：禽流感是一个全球性的公共卫生威胁。了解屠宰者所具备的禽流感知识和他们所采取的预防措施是设计未来备灾项目的关键。本研究旨在确定尼泊尔加德满都地区屠宰者缺乏禽流感知识及预防措施的社会因素。

方法：本研究基于横断面研究设计，采用结构化问卷面试和检查清单调查以了解加德满都地区的 120 个 15 岁以上屠宰人员的社会因素和预防措施。

结果：大部分受访者为男性（69.2％），25-39 岁年龄组（31.08±9.82 岁）的人数超过受访者的一半（53.3％）。近 2/3 的受访者（61.3％）禽流感知识贫乏，其余对禽流感有一些了解。超过一半的受访者（55.4％）对禽流感的预防措施不足，剩下的则归入实践满意的范畴。没有一位受访者有足够的禽流感知识或采取了令人满意的预防措施。与<25 岁年龄组相比，>25 岁的受访者所具备的禽流感知识更多[OR 0.169，95％CI（0.056-0.512）]。与接受过中学或以上教育的受访者相比，仅受过小学教育的受访者的禽流感知识更为贫乏[OR 3.265，95％CI（1.326-8.189）]。不知道禽流感的正确定义[OR 4.265，95％CI（1.193-15.242）]和不知道哪些是高危人群[OR 3.103，95％CI（1.191-8.083）]的受访者更可能预防措施缺失。

结论：本研究指出需要促使屠宰者提高他们的知识水平，更重要的是，需要改善他们对预防措施的依从性以预防禽流感。

Translated from English version into Chinese by Yang Pin, through

CHINESE CENTER FOR DISEASE CONTROL AND PREVENTION
NATIONAL INSTITUTE FOR PARASITIC DISEASES

Déterminants sociaux donnant lieu à une mauvaise connaissance de la grippe aviaire et à des pratiques de précaution inappropriées chez les bouchers de Katmandou, au Népal

Mohan Paudel, Bimala Acharya et Mandira Adhikari

Résumé

Contexte : La grippe aviaire (GA) est une menace mondiale pour la santé publique. Il est crucial, pour la conception des programmes de préparation à venir, de comprendre la connaissance des bouchers et leurs pratiques de précaution à ce sujet. Cette étude visait à identifier les déterminants sociaux de la connaissance et des mesures de précaution envers la GA chez les bouchers du district de Katmandou, au Népal.

Méthodes : L'étude a été basée sur un concept transversal utilisant des listes de critères et des questionnaires structurés pour observer les déterminants sociaux et les mesures de précaution de 120 bouchers âgés de 15 ans et plus du district de Katmandou.

Résultats : La majorité des personnes interrogées étaient des hommes (69,2%) et plus de la moitié (53,3%) appartenaient au groupe d'âge des 25-39 ans (moyenne : 31,08, DS : ± 9,82). Près des deux tiers (61,3 %) des personnes interrogées avaient une « mauvaise connaissance », et les autres avaient « une certaine connaissance » de la GA. Plus de la moitié (55,4%) des personnes interrogées étaient dans la catégorie des « mauvaises pratiques » envers la GA et l'autre moitié étaient dans la catégorie des « pratiques satisfaisantes ». Aucune des personnes interrogées n'avait une « connaissance suffisante » ni de « bonnes pratiques ». Les personnes interrogées du groupe d'âge > 25 ans étaient moins susceptibles [RR 0,169, IC à 95 % (0,056-0,512)] par rapport à celles du groupe d'âge < 25 ans d'avoir une mauvaise connaissance de la GA, et les personnes interrogées ayant une « éducation primaire » étaient plus susceptibles [RR 3,265 ; IC à 95% (1,326-8,189)] d'avoir une mauvaise connaissance de la GA par rapport à celles ayant une éducation de niveau secondaire ou supérieur. Les personnes interrogées qui ne connaissaient pas la bonne définition de la GA étaient plus susceptibles d'avoir de mauvaises pratiques [RR 4,265 ; IC à 95 % (1,193-15,242)], et les personnes interrogées qui ne connaissaient pas les groupes de risque associés à la GA étaient également plus susceptibles d'avoir de mauvaises pratiques [RR 3,103 ; IC à 95 % (1,191-8,083)].

Conclusion : Cette étude souligne la nécessité de prendre en considération les bouchers et d'améliorer leur connaissance des mesures de précaution pour prévenir la grippe aviaire et surtout leur conformité avec celles-ci.

Translated from English version into French by Jacek Sierakowski, through

TRANSLATORS
WITHOUT BORDERS

Социальные факторы, влияющие на недостаточную осведомленность торговцев мясом в Катманду (Непал) о птичьем гриппе и на ненадлежащий характер предпринимаемых ими мер, направленных на профилактику заболевания

Мохан Паудел , Бимала Ашаря и Мандира Адхикари

Краткий обзор

Предпосылки: Птичий грипп (ПГ) представляет собой угрозу населению всего земного шара. Поэтому для разработки дальнейших программ, направленных на борьбу с заболеванием, необходимо иметь представление о степени осведомленности торговцев мясом об этой болезни, а также о профилактических мерах, предпринимаемых ими для ее предотвращения. Целью настоящего исследования является определение социальных факторов, влияющих на осведомленность торговцев мясом региона Катманду (Непал) о птичьем гриппе, а также на предпринимаемые ими профилактические меры.

Методы: Исследование проводилось путем единовременного анкетирования с перечнем контрольных вопросов, направленное на выявление социальных факторов и профилактических мер, предпринимаемых 120 торговцами мясом в возрасте от 15 лет, проживающими в регионе Катманду.

Результаты: Большинство опрошенных были мужского пола (69,2%); возраст большей части (53,3%) составлял от 25 до 39 лет (средний возраст: 31,08 лет; стандартное отклонение: ±9,82). Приблизительно две трети (61,3%) опрошенных имели "слабое представление" о птичьем гриппе; остальные знали о нем "немного". Более половины опрошенных (55,4%) принадлежали к группе людей, "не предпринимающих надлежащих мер" по профилактике птичьего гриппа; меры, предпринимаемые остальными опрошенными меры были "удовлетворительными". Ни один из опрошенных не смог продемонстрировать "высокий уровень осведомленности" или "принятие надлежащих профилактических мер". Опрошенные в возрасте от 25 лет реже имели слабое представление о птичьем гриппе [относительный риск 0,169; 95% доверительный интервал (0,056-0,512)], чем опрошенные моложе 25 лет; опрошенные с начальным образованием чаще имели слабое представление о птичьем гриппе [относительный риск 3,265; 95% доверительный интервал (1,326-8,189)], чем опрошенные с образованием выше среднего. Опрошенные, которые не знали

определения птичьего гриппа, чаще всего не предпринимали надлежащих мер по его профилактике [относительный риск 4,265; 95% доверительный интервал (1,193-15,242)]; опрошенные, не имевшие представления о связанных с птичьим гриппом группах риска, чаще всего не предпринимали надлежащих мер по его профилактике [относительный риск 3,103; 95% доверительный интервал (1.191-8.083)].

Заключение: Исследование продемонстрировало необходимость информирования торговцев мясом о птичьем гриппе с целью улучшения их представления об этом заболевании, а также, что немаловажно, для принятия ими надлежащих профилактических мер для его предотвращения.

Translated from English version into Russian by Natalia Potashnik, through

TRANSLATORS WITHOUT BORDERS

Determinantes sociales que conducen a la falta de conocimientos acerca de las prácticas preventivas adecuadas relacionadas con la gripe aviar entre los carniceros de Katmandú, Nepal

Mohan Paudel, Bimala Acharya y Mandira Adhikari

Resumen

Antecedentes: La gripe aviar (GA) es una amenaza a la salud pública mundial. Entender el conocimiento que tienen los carniceros al respecto y las medidas de precaución que se toman en su contra es fundamental para el diseño de programas de preparación para el futuro. Este estudio tuvo como objetivo identificar los determinantes sociales del conocimiento y las medidas cautelares de la gripe aviar entre los carniceros del distrito de Katmandú en Nepal.

Métodos: El estudio se basó en un diseño de estudio transversal mediante cuestionarios de entrevistas estructuradas y listas de control para observar los determinantes sociales y las medidas cautelares de 120 carniceros de 15 años y más del distrito de Katmandú.

Resultados: La mayoría de los encuestados era de sexo masculino (69,2%) y más de la mitad (53,3%) pertenecía al grupo de 25 a 39 años de edad (media: 31,08, DE[1]: ± 9,82). Casi dos tercios (61,3%) de los encuestados tenía "escaso conocimiento" y el resto tenía "cierto conocimiento», sobre la GA. Más de la mitad (55,4%) de los encuestados se encontraba en la categoría que daba muestras de "mala práctica" hacia la GA y la otra mitad se encontraba en la categoría de "práctica satisfactoria”. Ninguno de los encuestados tenía un "conocimiento adecuado" o daba muestras de "buena práctica". Los encuestados en el grupo de más de 25 años de edad tenían menos probabilidades [OR 0,169, IC 95% (0,056-0,512)] comparados con los del grupo de menores de 25 años de tener un escaso conocimiento de la GA, y los encuestados con "educación primaria" tenían más probabilidades [OR 3,265, IC 95% (1,326-8,189)] de tener un escaso conocimiento de GA comparados con los que tenían un nivel de educación secundario o superior.

Los encuestados que no sabían la definición correcta de GA eran más propensos a seguir las prácticas deficientes [OR 4,265, IC 95% (1,193 a 15,242)], y los encuestados de los grupos

---

[1] DE: datos de la encuesta

que no conocían los riesgos asociados con la GA también eran más propensos a seguir malas prácticas [OR 3,103, IC del 95% (1,191 a 8,083)].

Conclusión: Este estudio señala la necesidad de abordar a los carniceros para mejorar su conocimiento y, lo que es más importante, su observancia de las medidas cautelares para prevenir la gripe aviar.

Translated from English version into Spanish by Andrea Ali, through

TRANSLATORS
WITHOUT BORDERS
